# Surface-Ligand-Modified CdSe/CdS Nanorods for High-Performance Light-Emitting Diodes

**DOI:** 10.1021/acsomega.2c05730

**Published:** 2023-01-19

**Authors:** Hui Zhang, Xiaohu Mi, Bowen Kang, Yunkai Wu, Tingting Zhang, Pai Liu, Xiaowei Sun, Zhenglong Zhang, Ning Liu, Hongxing Xu

**Affiliations:** † Department of Physics and Bernal Institute, University of Limerick, Limerick V94 T9PX, Ireland; ‡ School of Physics and Information Technology, Shaanxi Normal University, Xi’an 710119, China; § College of Big Data and Information Engineering, Guizhou University, Guiyang 550025, China; ∥ Institute of Nanoscience and Applications, Southern University of Science and Technology, Shenzhen 518055, China; ⊥ School of Physics and Technology, Wuhan University, Wuhan 430072, China; # Key Lab of Acupuncture and Drug Combination, Shaanxi University of Traditional Chinese Medicine, Xianyang 712044, China

## Abstract

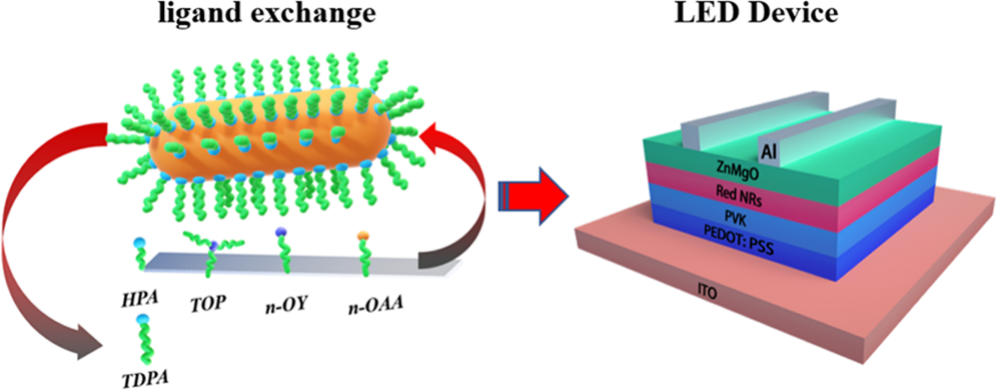

Colloidal nanocrystals (NCs) play an important role in the field of optoelectronic devices such as photovoltaic cells, photodetectors, and light-emitting diodes (LEDs). The properties of NC films are strongly affected by ligands attached to them, which constitute a barrier for charge transport between adjacent NCs. Therefore, the method of surface modification by ligand exchange has been used to improve the electrical conductivity of NC films. However, surface modification to NCs in LEDs can also affect emission characteristics. Among NCs, nanorods have unique properties, such as suppression of nonradiative Auger recombination and linearly polarized light emission. In this work, CdSe/CdS nanorods (NRs) were prepared by the hot injection method. To increase the charge transport into CdSe/CdS NRs, we adopted ligand modification to CdSe/CdS NRs. Using this technique, we could shorten the injection barrier length between CdSe/CdS NRs and adjacent layers. It leads to a more balanced charge injection of electron/hole and a greatly increased current efficiency of CdSe/CdS NR-LEDs. In the NR-LEDs, the ligand exchange boosted the electroluminance, reaching a sixfold increase from 848 cd/m^2^ of native surfactants to 5600 cd/m^2^ of the exchanged *n*-octanoic acid ligands at 12 V. The improvement of CdSe/CdS NR-LED performance is closely correlated to the efficient control of charge balance via ligand modification strategy, which is expected to be indispensable to the future NR-LED-based optoelectronic system.

## Introduction

Semiconductor quantum dots, as a promising material for high-efficiency light-emitting diodes (LEDs), have been widely used for optoelectronic devices due to their unique optical properties such as narrow-bandwidth emission, wide emission wavelength tunability, and high photoluminescence quantum yield (PLQY).^1−6^ Colloidal quantum dots (cQDs), without the shape limitation from lithography, can cover the whole visible as well as the near-infrared spectral range by flexibly varying the size of the nanocrystals, which shows a greater potential in full-spectrum modulation.^7^ Compared to epitaxial quantum dots, cQDs typically have a severe dielectric discontinuity across the surface, resulting in a significant increase in the Coulombic interaction^8−10^ as well as exciton binding energy.^11,12^ The increased Coulombic interaction and exciton binding energy of cQDs will contribute to higher optical quality with appropriate excitation. Moreover, the chemical and optical properties of cQDs can be changed by modifying different ligands on their surfaces, which not only can be used in high-brightness multicolor displays like laser TVs but also for lasers, LEDs, and solar concentrators due to their broad spectrum tunability and low emission linewidth.^13−15^

As an emerging class of fluorescent materials, the development of nanorods (NRs) with heterostructure-based LEDs is still relatively limited compared to the traditional spherical QDs. Like spherical QDs, the NR with a heterostructure has a wide tunable range of emission wavelengths by the modulation of the diameter or width of the NRs or by varying the core diameter in the core–shell NRs. Semiconductor NR has several unique optical properties, such as linearly polarized emission, a larger Stokes shift, a faster radiative decay process, and slower bleaching kinetics than spherical QDs. These advantages demonstrate that nonspherical NRs have good prospects in the next-generation display and lighting applications.

The external quantum efficiency (EQE) of a LED, the ratio of the number of output photons to the number of injected electrons, is an essential LED parameter. Some factors can significantly influence the EQE of a LED, such as the charge carrier mobility, injection rate, optical out-coupling, and PLQY of the active NR layer. The presence of ligands on the surface of NRs has a major impact on the electrical properties of the NRs, as well as the device’s performance. The ligands passivate the surface of the NRs, changing the density and depth of trap states and, as a result, charge transport.^16^ The charge carrier mobility of the film is normally characterized by the surface ligands, which act as tunnel barriers for charge transfer between two NRs.^17^ Surface ligands are critical for colloidal stability and solution processability in NR synthesis. Bulky ligands like trioctylphosphine oxide (TOPO) and octadecylphosphonic acid (ODPA) are excellent at this task; however, they are not suitable for optoelectronic applications. As a result, strategies have been devised to replace these long-chain ligands with shorter ones.^18,19^ This type of ligand-exchange treatment on colloidal QDs has been used to increase the performance of electroluminescence (EL) devices. High mobility leads to efficient exciton generation in devices that rely on the forward injection of electrons and holes, such as LEDs. However, implementing such techniques of ligand exchange to QD-based LEDs is difficult, since they often degrade the PLQY, which has a negative impact on the device’s internal quantum efficiency. Therefore, optimized techniques of ligand exchange as well as a thorough understanding of the impact of surface ligands on LED performance are critical for the development of NR-based LEDs.

In this work, we developed a CdSe/CdS nanorod (NR)-based NR-LED device with high photoluminescence quantum yield, improved EL, and good stability through surface ligand modifications. The influence of the NRs’ performances from different surface ligand modifications is investigated in detail. In the NR-LED device, the ligand exchange boosted the luminance, reaching a sixfold increase from 848 cd/m^2^ of native surfactants to 5600 cd/m^2^ of the exchanged *n*-octanoic acid ligands at 12 V. This study could pave the way for a new generation of lighting systems with great color purity, processability, and stability.

## Results and Discussion

**Figure 1:**
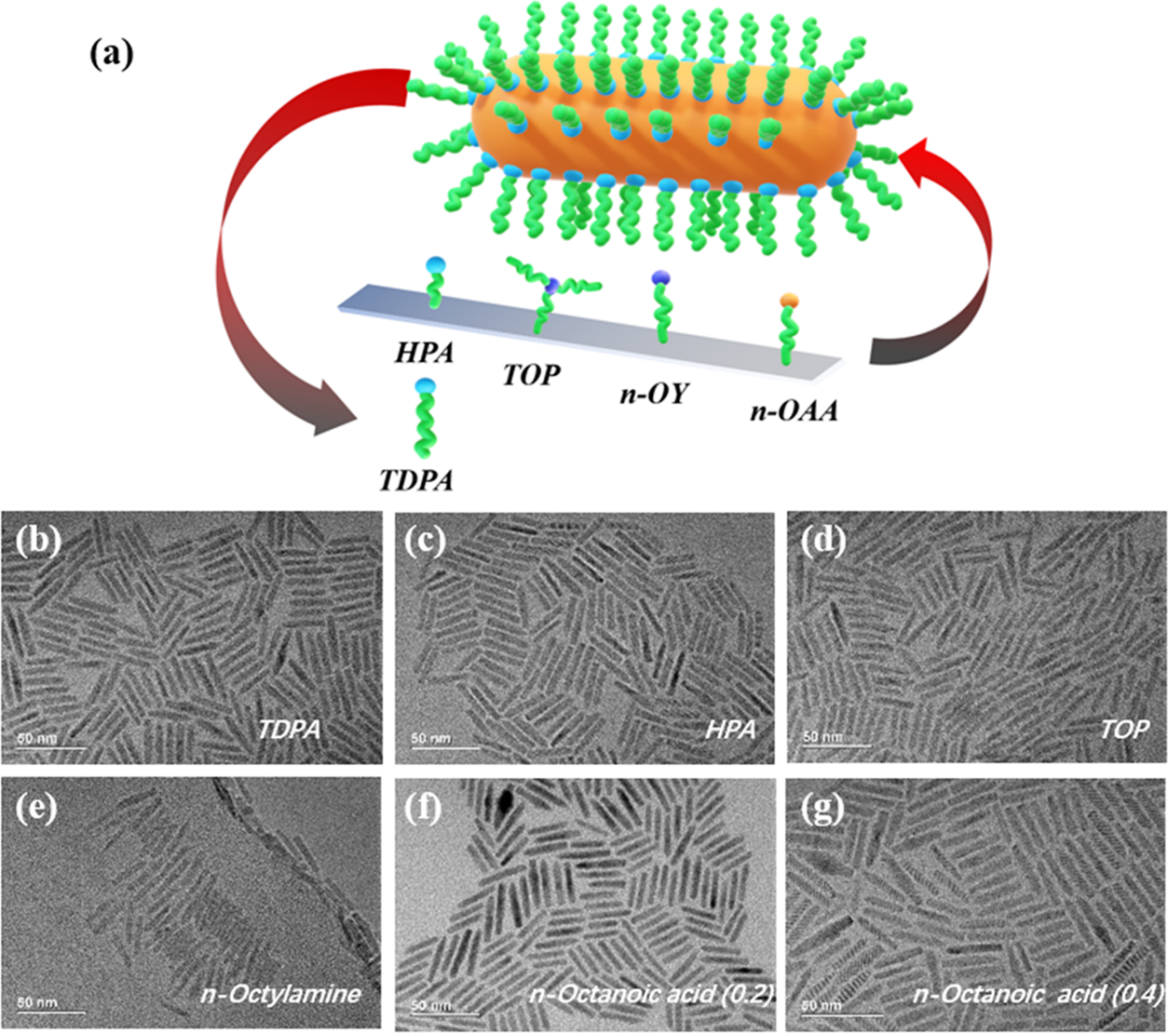
(a) Schematic diagram of the exchange of different ligands. (b) TEM image of original CdSe/CdS NRs (TDPA). (c) TEM image of CdSe/CdS NRs (HPA). (d) TEM image of CdSe/CdS NRs (TOP). (e) TEM image of CdSe/CdS NRs (*n*-octylamine). (f, g) TEM images of CdSe/CdS NRs (*n*-octanoic acid of different concentrations, see Materials and Methods).

The original CdSe/CdS NRs are synthesized via the seeded growth method according to the previous report.^20−22^ In a typical synthesis of CdSe/CdS NRs, the length-to-diameter ratios can be altered by adjusting the size of the core and the proportion of the shell precursor. The size of the CdSe core could be adjusted by altering the temperature, reaction time, and type of phosphonic acid (ODPA or TDPA). The relative binding energies of ligands to different facets determine the growth rates of the different facets and, as a result, control the geometry of the resultant nanoparticles.^23,24^ Herein, CdSe/CdS core–shell NRs with 30 nm length are successfully synthesized via the above-mentioned method. By adding different chemical reagents to the synthetic stock solution, the exchange of different ligands can be achieved (Figure 1a). Since we cannot accurately predict the influence of different ligands on the nanorods after the exchange, the principle of ligand selection in the current work is as follows: (1) functional group, (2) structure, and (3) chain length. As a result, we choose phosphate, phosphine, amino, and carboxyl groups. We have branched and straight chains. In the straight chain, we also have different chain lengths. Five different surface ligands including the original TDPA, *n*-hexyl phosphonic acid (HPA), trioctylphosphine (TOP), *n*-octylamine, and *n*-octanoic acid are chosen for investigating the optical properties of CdSe/CdS NRs, where the corresponding transmission electron microscopy (TEM) images are shown in Figure 1b–g (see Materials and Methods for synthesis details).

**Figure 2:**
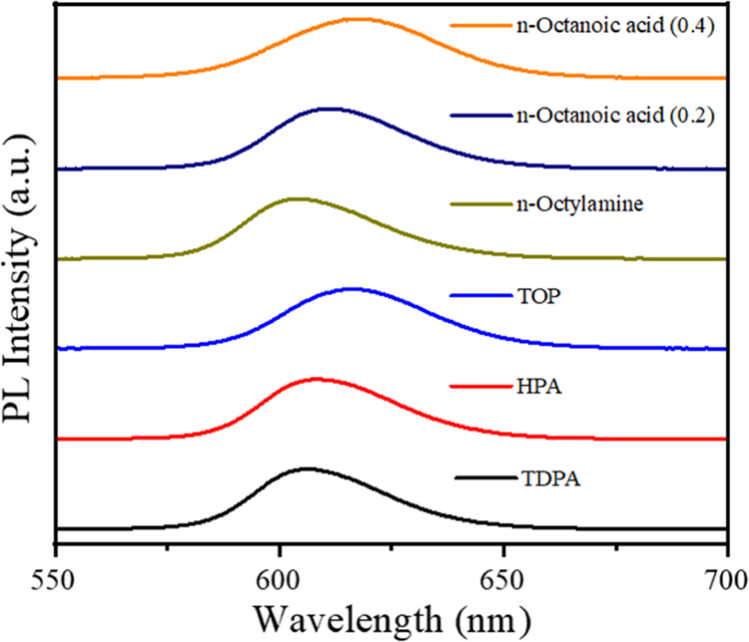
Normalized photoluminescent spectra of the laser-excited CdSe/CdS NRs with different surface ligand modifications.

First, we investigated the photoluminescent (PL) properties of CdSe/CdS NRs modified by different ligands (more information can be found in Materials and Methods) As shown in Figure 2, compared with the original TDPA-terminated NRs, the *n*-octylamine-modified NRs show an obvious blue-shift of the PL peak, whereas the HPA-, TOP-, and *n*-octanoic acid-modified NRs exhibit varying degrees of red-shift. This is mainly caused by the change of the surface charge and defects of the NRs caused by the surface ligand exchange, which leads to the shift of the energy band. The band alignment modification could explain the blue-shift of the *n*-octylamine-modified NRs in the optical spectrum: The surface ligands exchange can influence the band alignment. The band gap widens, leading to the emission wavelength shift. Similarly, the red-shifts of PL peaks for the HPA-, TOP-, and *n*-octanoic acid-modified NRs are due to the slight aggregation of the CdSe/CdS NRs, resulting in the band gap shrinking. The laser-excited PL spectra demonstrate that the ligand can directly affect the surface defects of the NRs, leading to the emission shift of the PL spectra.

**Figure 3:**
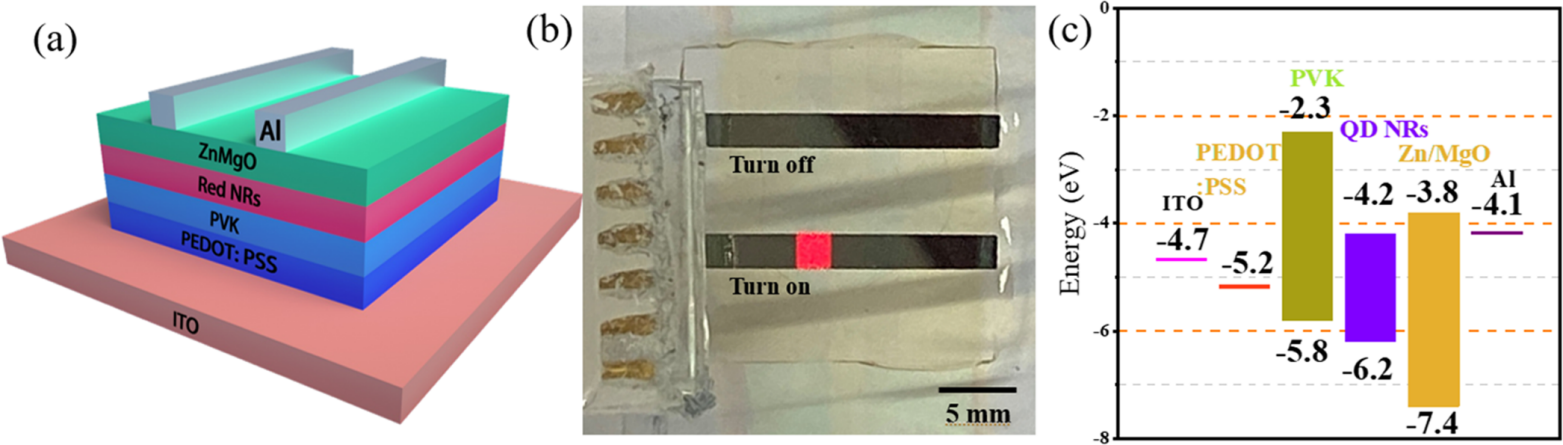
(a) Schematic structure of red NRs EL-QLEDs. (b) Photograph of NR-LED. (c) Flat-band diagram of the EL-NR-LED structure.

The electrically driven performances of the CdSe/CdS NRs modified by different ligands are further investigated. Typical EL of NR-LEDs with the structure of indium tin oxide (ITO)/poly(3,4-ethylenedioxythiophene):polystyrene sulfonate (PEDOT:PSS)/poly(9-vinlycarbazole) (PVK)/NRs/ZnMgO/Al were produced to test the effect of CdSe/CdS NRs modified by different ligands on device performance. The device construction is depicted schematically in Figure 3a. ZnMgO (electron transport layer) aids to inhibit exciton dissociation at the interface of NRs and metal oxides by widening band gaps and lifting conduction band minima. After applying the external voltage to this device, bright red fluorescence is emitted from the device, which can be clearly observed in the picture shown in Figure 3b. Furthermore, the band energy levels of each material from NR-LED structure are shown in Figure 3c. The band energy levels of CdSe/CdS NRs, electron/hole transport layers, are obtained from the previously reported works.^25,26^

**Figure 4:**
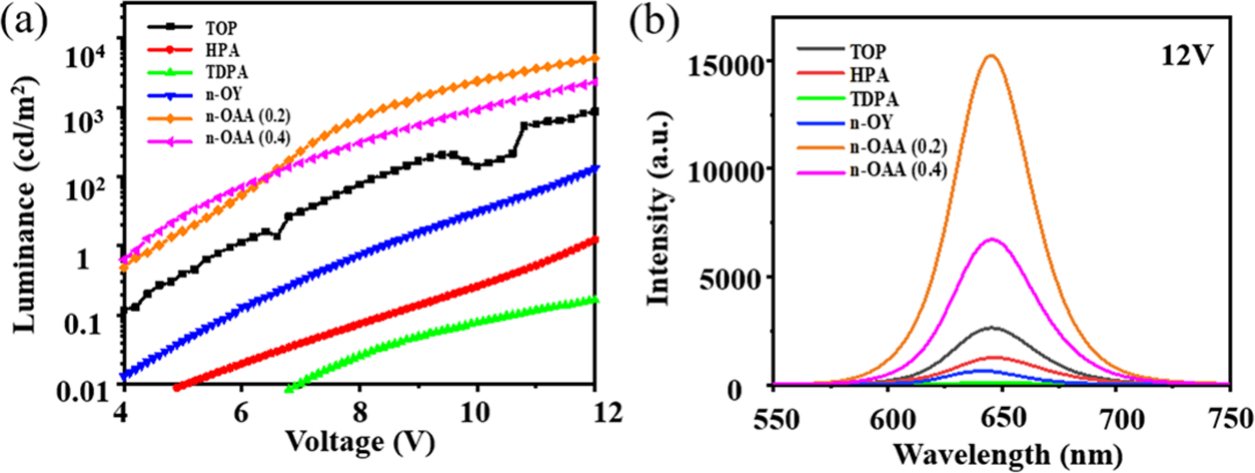
(a) Luminance intensity vs voltage characteristics of EL-NR-LEDs based on NRs modified by different ligands. (b) EL spectra of NRs modified by different ligands under 12 V voltage. Here, *n*-OY and *n*-OAA correspond to *n*-octylamine and *n*-octanoic acid, respectively.

Figure 4 shows the properties of the EL of NR-LED with NRs modified by different ligands. The luminance–voltage characteristics of NR-LEDs with varied ligand-adjusted NRs are comparable to the reference device in Figure 4a. The luminance, on the other hand, is substantially boosted at high driving voltages for *n*-octanoic acid-modified NRs compared to the reference device. The maximum luminance for NR-LED with *n*-octanoic acid (V./2:10)-modified NRs reaches 5600 cd/m^2^ at 12 V, which is significantly higher than 848 cd/m^2^ for the CdSe/CdS NR reference device, implying that *n*-octanoic acid can effectively improve device efficiency. Figure 4b shows the steady EL measurement results under specific voltage (12 V) excitation. Similarly, the EL peaks also show a small shift due to the charge carrier mobility change resulting from the surface defect altered by the ligand. The *n*-octanoic acid-modified NRs show extraordinary performance in the steady-state test.

**Figure 5:**
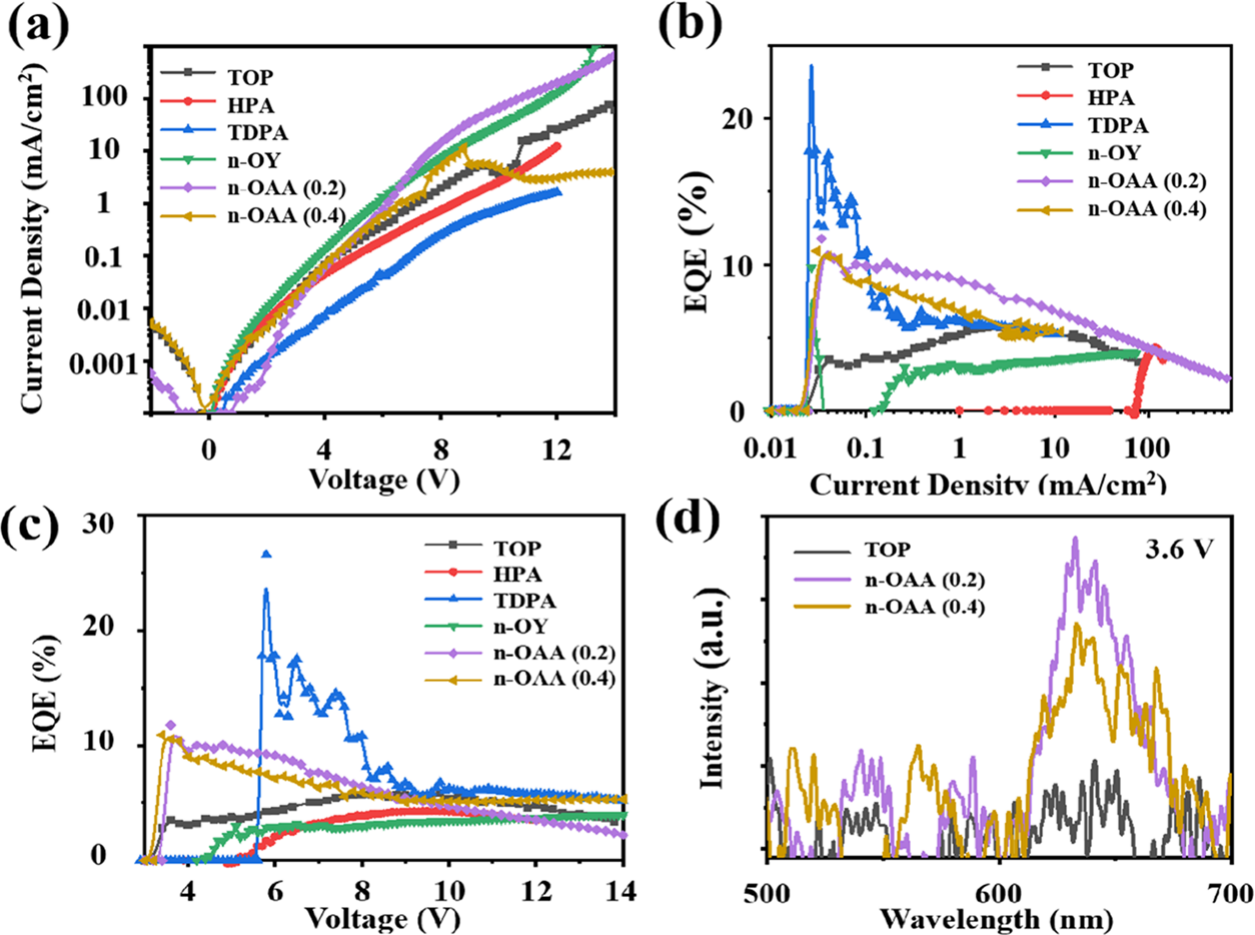
(a) Current density vs voltage characteristics of EL of NR-LEDs based on NRs modified by different ligands. (b) EQE vs current density characteristics of NRs modified by different ligands. (c) EQE vs voltage characteristics of NRs modified by different ligands. (d) EL spectra of NRs modified by different ligands under a low voltage of 3.6 V. Here, *n*-OY and *n*-OAA correspond to *n*-octylamine and *n*-octanoic acid, respectively.

Figure 5 summarizes the performance of electrically pumped NR-LED devices with NRs modified by different ligands. As shown in Figure 5a, we can see the effect of improved charge carrier transport after ligand exchange. For the TDPA (14-carbon chain)-terminated NR-based device, the current is the smallest at a specific applied voltage compared to other devices. All other devices present an improved current density. Specifically, the *n*-octanoic acid (V./2:10)-modified NR-based device shows an excellent sensitivity of the current–voltage response compared with other ligand-modified NR-based devices, an indication of better charge transport to allow the recombination of carriers. Figure 5b,c show the EQE variation with the current density and the input voltage, respectively. Although the TDPA-terminated NR-LED device exhibits good EQE performance at the low current density and voltage, which can be attributed to a smaller leakage current at low current density owing to its long carbon chain, the fast decay rate of the EQE with the increase in current as well as voltage demonstrates the unstable performance of the NR-LED device with TDPA-modified NRs. Both *n*-octanoic acid-modified NRs with different volume ratios indicate a stable performance on the EQE test. Under the condition of low applied voltage, only the NR with the *n*-octanoic acid exchange can achieve a larger EQE. The EQE roll-off improvement can also be observed in Figure 5b. The device made from NR with *n*-octanoic acid exchange (0.2) demonstrates overall the best EQE roll-off with the increase of the injection current compared to other devices, which indicates a more stable device performance.

Last but most importantly, ligand-modified devices can be triggered at a very low threshold voltage. As shown in Figure 5d, the luminescence can be observed at an input voltage of 3.6 V, which means the turn-on voltage is reduced by improved charge injection.

## Conclusions

In summary, the performance of NR-LEDs based on CdSe/CdS NRs can be obviously improved by ligand exchange. Such ligand-exchange processes can improve the efficiency and stability of nanocrystal-based LEDs fabricated from these ligand-modified rice-shaped nanorods. In this work, a luminosity enhancement can be obtained from 848 cd/m^2^ for TDPA ligands to 5600 cd/m^2^ for devices passivated with *n*-octanoic acid, which shows a significant reduction in the efficiency roll-off. The fundamental properties of CdSe/CdS NRs with diverse ligands are examined and some insights into their impact on device performance are discussed. The result demonstrates that the surface chemistry of that material is of crucial importance for the final performance of LEDs.

## Materials and Methods

### Synthesis of CdSe/CdS NRs

Synthesis of CdSe/CdS nanorods has been carried out following similar methods of previous reports.^20,21^ To synthesize the CdSe core, 78 mg of cadmium oxide (CdO, 99.99%), 4.5 g of trioctylphosphine oxide (TOPO, 99%), and 0.336 g of tetradecylphosphonic acid (TDPA, 97%) were added in a 50 mL three-necked flask. The mixture was then heated to 150 °C, and the argon gas was filled for 1 h. Next, when the temperature reached 300 °C, the solution changed to clear and colorless, which meant that the reaction between CdO and TDPA was completed. Then, when the temperature increased to 325 °C, 2.25 mL of trioctylphosphine (TOP, 97%) was injected into the flask. Subsequently, once the mixture was heated to 370 °C, 0.95 mL of Se/TOP stock solution (1 mol/L) was quickly injected into the three-necked flask, which was kept at this temperature for 20 s. To synthesize the CdS shell, the mixture of 57.9 mg of CdO, 81 mg of *n*-hexyl phosphonic acid (HPA, 97%), 3 g of TOPO, and 0.3 g of TDPA was first heated to 150 °C and exposed to argon for 1 h. TOP (1.5 mL) was then injected into the mixture when the temperature reached 300 °C. The mixture of 1.5 mL of S/TOP (2.0 mol/L) and 0.425 mL of the as-synthesized CdSe core solution was injected into the flask quickly and kept at 320 °C for 8 min. Lastly, the solution was cooled to room temperature, purified, and collected for further surface ligand modification.

### Ligand Exchange in Solution

The ligands, HPA (0.1mol/l, in methylbenzene), TOP, *n*-octylamine, and *n*-octanoic acid, were mixed with the as-synthesized CdSe/CdS NRs (9 mg/mL) and then dispersed in *n*-hexane in a ratio of V./1:1, V./2:10, V./2:10, and V./2:10/V./4:10, respectively. The solution was vibrated on an oscillator for 20 min and added with ethanol. Next, the solution was centrifuged at 10,000 rpm and kept for 3 min. Finally, the NRs were redispersed in 1 mL of *n*-hexane for further characterization.

### LED Device Fabrication and Characterization

Red NR-LEDs were prepared on patterned ITO glass substrates, which were first cleaned in an ultrasonic bath. To verify the positive effects of ligand-exchanged CdSe/CdS NRs on device performance, NR-LEDs with the device structure of ITO/PEDOT:PSS//PVK(poly)/NRs/Zn_*x*_Mg_1–*x*_O NPs/Al were fabricated, in which PEDOT:PSS was spin coated on ITO glass with 3000 rpm for 45 s, and then baked at 130 °C for 15 min. PVK dissolved in chlorobenzene (8 mg mL^–1^) was spin coated at 3000 rpm, followed by being baked at 120 °C for 10 min. Then, CdSe/CdS nanorods from a 9 mg mL^–1^ solution were spin coated at 3000 rpm for 45 s, followed by 5 min annealing at 100 °C. Next, Zn_*x*_Mg_1–*x*_O was spin coated from a solution of 20 mg mL^–1^ in ethanol at 3000 rpm, followed by annealing at 100 °C for 45 s. Lastly, the 100 nm thick Al was deposited by thermal evaporation in a vacuum chamber.

The EL spectrum of NR-LEDs was measured by a fiber optic spectrometer (Ocean Optics USB 2000) from the EL area. In this test system, a 4 mm^2^ emitting area was considered a Lambeau luminaire. The luminance can be calculated using the human visual photonic curve by combining the luminous power and the EL spectrum. The current density–luminance–voltage curves of red NR-LEDs were measured by a dual-channel Keithley 2614B source meter and a PIN-25D silicon photodiode in ambient conditions. Table 1 below summarizes the PL (Figure 2) and EL (Figure 4b) emission maxima and FWHM, along with the PLQY.

**Table 1:** 

ligand	PL emission maximum (nm)	PL FWHM (nm)	PLQY (%)	EL emission maximum (nm)	EL FWHM (nm)
TOP	616.5	37.5	40.5	648.3	43.3
HPA	609.1	34.8	65.3	649.6	46.1
TDPA	606.1	33.4	84.1	649.5	41.4
*n*-OY	604.6	34.5	85.3	644.0	38.9
*n*-OAA (0.2)	611.3	35.0	76	646.7	40.7
*n*-OAA (0.4)	618.0	41.0	32.6	648.4	43.6

### Transmission Electron Microscopy

CdSe/CdS nanorods were drop cast from a diluted solution onto 200-mesh carbon-coated copper grids. A JEOL JEM-1011 microscope was used to obtain the TEM images at a 100 kV accelerating voltage.
